# Strengthening diabetes education and peer support in South Africa: Insights from a national stakeholder convening ahead of the 2025 Diabetes Summit

**DOI:** 10.4102/phcfm.v18i2.5572

**Published:** 2026-07-31

**Authors:** Lorrein S. Muhwava, Patrick Ngassa Piotie, Kibachio J. Mwangi, Melanie Pienaar

**Affiliations:** 1 Noncommunicable Diseases Programme, Foundation for Innovative New Diagnostics (FIND), Geneva, Switzerland; 2 School of Health Systems and Public Health, Faculty of Health Sciences, University of Pretoria, Pretoria, South Africa; 3 Department of Public Health, Medical School, Nelson Mandela University, Gqeberha, South Africa; 4 Noncommunicable Diseases and Mental Health, Health Promotion, Disease Prevention and Control, World Health Organization, Pretoria, South Africa; 5 School of Nursing, Faculty of Health Science, University of the Free State, Bloemfontein, South Africa

## Introduction

Diabetes mellitus remains one of the rapidly increasing noncommunicable diseases (NCDs) in South Africa, contributing substantially to morbidity, premature mortality and increasing health system costs.^1,2^

Despite the availability of effective pharmacological therapies, outcomes for many people living with diabetes mellitus remain suboptimal, driven in part by poor glycaemic control, late diagnosis and preventable complications, such as amputations, kidney failure, cardiovascular disease and vision loss.^3,4^ These challenges are particularly pronounced in the public health sector, where primary health care (PHC) services are overstretched, and clinical encounters often leave little time for structured patient education or ongoing self-management support.^5^

The 2023 South African Diabetes Summit identified diabetes education and self-management support as the most critical gaps in the national diabetes response.^6^ The Summit noted the absence of a structured, standardised national diabetes self-management education (DSME) programme, the limited availability of diabetes nurse educators in the public sector and the resulting overreliance on brief, informal education during routine clinic visits as barriers to effective self-management and continuity of care. In this context, civil society organisations (CSOs) engaged in diabetes advocacy, education and peer support play an increasingly critical role in addressing gaps within the health system.

Before the 2025 Diabetes Summit, the Diabetes Alliance convened a national meeting of key stakeholders, including CSOs involved in diabetes advocacy, national and provincial government representatives, and the World Health Organization (WHO), to collectively reflect on the state of diabetes education and peer support in South Africa. This conference report synthesises the key insights emerging from the workshop and considers their implications for diabetes policies and practices.

## The civil society convening: Purpose, participants and process

The convening was organised by the Diabetes Alliance in partnership with the National Department of Health (DOH) and the WHO as part of the preparatory activities for the 2025 Diabetes Summit. Its primary aim was to create a dedicated space for civil society actors to reflect on diabetes education and peer support following the 2023 Summit, in which these issues were identified as priority areas for action.

The workshop participants included representatives from the National DOH, WHO, Gauteng DOH and CSOs involved in diabetes education, peer support, community outreach and advocacy. Many participants had lived experiences of diabetes mellitus, either personally or through close engagement with communities affected by the condition. This diversity of perspectives was intentionally leveraged to foreground experiential knowledge alongside organisational and programmatic insights.

The meeting was structured around facilitated group discussions, allowing participants to share their experiences, identify challenges and propose solutions related to diabetes education and peer support. In this report, a peer refers to a person with lived experience of diabetes, either as someone living with the condition or as an individual closely engaged with diabetes-affected communities, who uses this experiential knowledge to support others. Peer support refers to structured or informal support provided by such individuals to people living with diabetes, including practical self-management guidance, emotional support, shared problem-solving, encouragement and linkage to information or services. The authors drew on discussion notes and collective reflections from the meeting to summarise the key themes and priorities.

## Key themes emerging from the discussions

### Fragmentation and inconsistency of diabetes education

The dominant theme across discussions was the fragmented and inconsistent nature of diabetes education in South Africa. Participants described a landscape in which education was delivered unevenly across provinces, facilities and sectors (public vs. private), with little coordination or standardisation. In many public sector settings, diabetes education is limited to brief verbal advice provided during clinical consultations, often without written materials, follow-up or adaptation to patients’ social and cultural contexts.

Civil society actors noted that this inconsistency contributes to confusion among people living with diabetes mellitus, undermines self-management and exacerbates inequities in care. Individuals with access to private care or well-resourced NGOs may receive comprehensive education and support, whereas others rely solely on overstretched clinics with limited capacity for patient education.

### Absence of a structured national framework for diabetes self-management education

Closely linked to fragmentation was the perceived absence of a *nationally endorsed framework for diabetes self-management education*. Participants emphasised that, unlike conditions such as human immunodeficiency virus or tuberculosis, diabetes mellitus lacks a clearly articulated education pathway within the public health system.^7^ There is no standard curriculum, formal referral mechanism to education services, or routine integration of education into chronic care pathways.

This gap places additional pressure on CSOs to develop their own educational materials and programmes, often with limited resources and variable levels of clinical oversight. While many organisations have developed innovative and contextually appropriate approaches, participants expressed concern that the lack of national guidance limits scalability and sustainability.

### The value of peer support grounded in lived experience

Civil society actors highlighted peer support as a particularly valuable component of diabetes education. Participants described peer support as uniquely positioned to address not only knowledge gaps, but also the emotional, social and practical challenges of living with diabetes mellitus.^8,9^ Shared lived experiences were seen as fostering trust, relatability and sustained engagement in ways that clinical interactions alone often cannot.

Peer support was described as taking multiple forms, including group education sessions, community-based support groups, one-to-one mentoring and increasingly, digital platforms such as messaging groups and online forums.^10,11^ Participants emphasised that peer support helps normalise the experience of diabetes mellitus, reduces isolation and distress and builds confidence in self-management.^12^

### Tensions between informal peer support and formal health systems

Despite strong enthusiasm for peer support, discussions highlighted tensions between informal peer-led initiatives and the formal health system. Participants noted that peer support programmes often operate in parallel to clinical services, with limited recognition, referral pathways or supervision from health professionals. This separation can lead to mistrust, duplication of efforts and missed opportunities for integration.

Some participants expressed concerns that without appropriate training and oversight, peer educators might feel ill-equipped to address complex clinical questions or complications. Simultaneously, overly rigid professional control was seen as potentially undermining the relational strengths that make peer support effective.

### Sustainability and capacity constraints within civil society

Finally, the participants highlighted challenges related to sustainability and organisational capacity. Many peer support and educational initiatives rely on short-term donor funding and substantial amounts of uncompensated or minimally compensated time from peer supporters and community members. Burnout, high turnover, and limited opportunities for skill development were identified as risks to programme continuity.

Civil society actors emphasised that while CSOs can innovate and respond rapidly to community needs, long-term impact requires predictable funding, institutional support and stronger linkages with government programmes.

### Peer support as a strategic opportunity for South Africa

The insights from the convening underscore why civil society increasingly views peer support as a strategic opportunity to strengthen diabetes care in South Africa. Peer support closely aligns with the reality of living with a lifelong condition that requires daily self-management, motivation and psychosocial resilience.

International and local evidence suggests that peer support can improve diabetes knowledge, self-efficacy and self-management behaviours, and in some contexts, glycaemic outcomes.^13,14,15^ Importantly, peer-led models are often more cost-effective and scalable than specialist-led models, particularly in resource-constrained settings. In South Africa, peer support resonates strongly with existing PHC approaches, including the use of community health workers and ward-based outreach teams and broader commitments to community participation and person-centred care.

From a civil society perspective, peer support is not viewed as a replacement for professional diabetes education but as a complementary approach that can extend the reach of the health system, enhance the continuity of care and address psychosocial needs that are often overlooked in clinical settings.

## Implications for policy and practice

The workshop highlighted the need for greater visibility and structural recognition of diabetes education and peer support in South Africa’s diabetes response. Participants emphasised the importance of national-level dialogue on how peer support could be formally integrated into diabetes care pathways. This would require the development of standardised training frameworks, supervision structures and referral mechanisms, while ensuring that the relational and experiential strengths that underpin peer-led approaches are preserved.

These insights highlight the importance of moving beyond problem identification to concrete discussions on implementation. Civil society actors recognised the need to engage explicitly with questions of scale-up, sustainability and system integration, including how peer support models can align with national NCD strategies and PHC reforms.

More broadly, the convening underscored the role of civil society as an important site of knowledge generation. The experiential insights shared during the discussions provided perspectives that are often absent from formal policy processes but are critical for designing interventions that are responsive to the lived realities of people affected by diabetes mellitus.

## Conclusion

This convening provided a timely opportunity to reflect on diabetes education and peer support in South Africa. The discussions revealed widespread concerns about fragmented education provision, the absence of a national DSME framework and the sustainability of community-led initiatives. Simultaneously, they underscored a strong collective belief in the potential of peer support to strengthen diabetes self-management, particularly when grounded in lived experiences and community engagement.

These insights offer an important foundation for advancing more coordinated, equitable and person-centred approaches to diabetes education. Recognising and integrating peer support within the broader health system may be a critical step towards improving outcomes for individuals living with diabetes mellitus and reducing the long-term burden of the disease.
